# Prevalence of hypertension in adults living at altitude in Latin America and the Caribbean: A systematic review and meta-analysis

**DOI:** 10.1371/journal.pone.0292111

**Published:** 2023-10-12

**Authors:** J. Pierre Zila-Velasque, David R. Soriano-Moreno, Sebastian A. Medina-Ramirez, Fabricio Ccami-Bernal, Sharong D. Castro-Diaz, Andrea G. Cortez-Soto, Analis L. Esparza Varas, Jared Fernandez-Morales, Juan J. Olortegui-Rodriguez, Isabel P. Pelayo-Luis, Jessica Hanae Zafra-Tanaka

**Affiliations:** 1 Facultad de Medicina Humana, Universidad Nacional Daniel Alcides Carrion, Pasco, Peru; 2 Red Latinoamericana de Medicina en Altitud e Investigacion (REDLAMAI), Pasco, Peru; 3 Unidad de Investigación Clínica y Epidemiológica, Escuela de Medicina, Universidad Peruana Unión, Lima, Peru; 4 Facultad de Medicina, Universidad Nacional de San Agustin de Arequipa, Arequipa, Peru; 5 Sociedad Científica de Estudiantes de Medicina de Ica, Universidad Nacional San Luis Gonzaga, Ica, Peru; 6 Universidad Nacional de Trujillo, La Libertad, Peru; 7 Sociedad científica de estudiantes de medicina de la Universidad Nacional de Trujillo, Trujillo, Peru; 8 Escuela de Enfermería, Universidad Peruana Unión, Lima, Peru; 9 Escuela de Medicina, Universidad Científica del Sur, Lima, Peru; Hospital Guillermo Kaelin de la Fuente, PERU

## Abstract

**Objective:**

The objective of this systematic review and meta-analysis was to assess the prevalence of hypertension in populations living at altitude in Latin America and the Caribbean.

**Methods:**

We conducted a systematic search from January 1, 2000 to January 10, 2023 in Web of Science (WoS)/Core Collection, WoS/Medline, WoS/Scielo, Scopus, PubMed and Embase databases. We included studies that assessed the prevalence of hypertension in altitude populations (>1500 m.a.s.l.) and these were meta-analyzed using a random-effects model. To assess the sources of heterogeneity, we performed subgroup and meta-regression analyses.

**Results:**

Thirty cross-sectional studies (117 406 participants) met the inclusion criteria. Studies used different cut-off points. The prevalence of hypertension in the studies that considered the cut-off point of ≥ 140/90 mmHg in the general population was 19.1%, ≥ 130/85 mmHg was 13.1%, and ≥ 130/80 mmHg was 43.4%. There was a tendency for the prevalence of hypertension to be higher in men. In meta-regression analyses, no association was found between altitude, mean age, year of publication, risk of bias and prevalence of hypertension.

**Conclusion:**

The prevalence of hypertension in the altitude population of Latin America and the Caribbean is lower than that reported in populations living at sea level and lower than other altitude populations such as Tibetans.

**PROSPERO:**

CRD42021275229.

## Introduction

Hypertension is a chronic noncommunicable disease that affected approximately 1.27 billion adults in 2019, projecting to 1.56 billion by 2025, with this increase being greatest in developing countries [1, 2]. It is considered a major risk factor for heart attacks and strokes [3], affecting 8.4 million and 7 million Americans in 2016, respectively [4]. The latter two being the leading causes of mortality in the world [5], with 110 346 deaths due to heart attack and 146 383 deaths due to stroke in 2017 [4].

Hypertension has risk factors such as high body mass index (BMI), family history, low socioeconomic level [6], high hematocrit, among others [7–9]. In the case of altitude, acute hypoxia generates an increase in cardiac output that is counteracted by a decrease in peripheral vascular resistance (hypoxic vasodilatation) [10]. Thus, blood pressure varies little. On the other hand, it has been shown that in chronic exposures to altitude there is an increase in blood pressure, due to increased erythropoietin, resulting in increased blood viscosity (higher hematocrit), increased serum levels of angiotensin-2, entotelin-1, among others [11, 12].

A previous systematic review [11] studied the blood pressure level and prevalence of hypertension in adults living at altitudes >2400 meters above sea level (m.a.s.l.), including Tibetan and Andean populations, and found a prevalence greater than 30%. Likewise, another systematic review [13] evaluated the prevalence of hypertension in residents older than 15 years of age in Tibet (3000 to 4300 m.a.s.l), and found hypertension prevalence ranging from 23% to 56%. However, the findings of these reviews may not be generalizable to other populations such as the Andes, as there are phenotypic differences between the two populations [12]. Tibetan residents have a better ventilatory response to hypoxia, blood oxygen saturation, and lower levels of pulmonary vasoconstriction and hemoglobin concentration compared to residents of the Andes [11, 14, 15].

Latin America and the Caribbean have mountainous regions with altitudes higher than 1500 m.a.s.l., being the region of residence of 32 million people; specifically, 24 million at altitudes between 2500 and 2999 m.a.s.l. and 8 million at 3500–5000 m.a.s.l. [16]. The prevalence of hypertension may vary in this region due to the difference in the previously mentioned risk factors, genetic factors, rurality and altitude levels [7–9, 17–19]. Therefore, the objective of this systematic review and meta-analysis was to identify the prevalence of hypertension in adults living at altitude in Latin America and the Caribbean, using different cut-off points for the diagnosis of hypertension. These results could guide public health policies for the prevention and control of hypertension in populations living at altitude.

## Methods

This systematic review followed the Preferred Reporting Items for Systematic Reviews and Meta-Analyses (PRISMA) 2020 guidelines (S1 Table) [20]. The protocol was registered in PROSPERO, number CRD42021275229.

### Eligibility criteria

We included peer-reviewed cross-sectional and cohort studies that presented information on the prevalence of hypertension in adults older than 18 years residing at altitude (general population, primary care centers, or hospitals) conducted in Latin America or in the Caribbean region. We only included articles published since year 2000 to provide more current data. We considered that the altitude is higher than 1,500 (m.a.s.l.), taking into account that, according to the literature, from this limit onwards, physiological and systemic changes occur [21]. The following cut-off points were considered for the diagnosis of hypertension: 1) 140/90 mmHg as recommended by the European Society of Cardiology (ESC) and European Society of Hypertension (ESH) Working Group Guidelines; 2) 130/85 mmHg according to the American Heart and Diabetes Association; and 3) 130/80 mmHg according to the American College of Cardiology (ACC) and the American Heart Association (AHA) [22–24]. Studies with fewer than 100 participants and manuscripts that are not available in full text were excluded. In case of duplicate populations, only the study with more complete data was included.

### Literature search and study selection

We conducted a systematic search in six databases: Web of Science (WoS)/Core Collection, WoS/Scielo, WoS/Medline, Scopus, PubMed, and Embase from January 1, 2000 to January 10, 2023. No language restrictions were applied. The complete search for each database is available (S2 Table). We also reviewed the bibliographic references of the included studies for potentially eligible studies.

The studies were exported to the Rayyan Software program, where duplicates were removed manually [25]. Subsequently, two authors (ACS, JFM) reviewed the articles by titles and abstracts independently to identify potentially relevant articles for inclusion. A third author (SAMR) resolved discrepancies at this stage. The chosen studies went on to full-text review (JPZV, SCD). Likewise, this process was performed independently and discrepancies were resolved by a third author (DRSM).

### Data extraction

Four authors (JPZV, ACS, JOR, AEV) independently extracted the following data of interest using a Microsoft Excel sheet: author, year of publication, study design, country, sample size, age, sex, resident type, altitude level, hypertension cut-off point, blood pressure characteristics (mean systolic and diastolic blood pressure value), and prevalence of hypertension. Discrepancies were resolved in discussions involving the four authors in charge of data extraction.

### Risk of bias

Four authors (SCD, JFM, IPL, FCB) independently assessed the methodological quality of prevalence studies using the Joanna Briggs Institute Critical Appraisal Tool [26]. Another author (SAMR) resolved discrepancies in this process. This scale has 9 items with possible responses of "Yes", "No" and "Unclear". The quality score presented in Table 1, was considered as one point to "Yes" and as zero points to "No" and "Unclear". In addition, we categorized the risk of bias into high, moderate, and low according to scores of 0 to 3, 4 to 6, and 7 to 9, respectively.

**Table 1:** Characteristics of included studies assessing the prevalence of hypertension in the altitude of Latin America and the Caribbean (n = 30).

Author—year	Country	Setting and population	Sample size	Altitude level (m.a.s.l)	General Characteristics of population				Hypertension definition	Hypertension prevalence (%)
					Age (mean ± SD years)	male (%)	SBP (mean ± SD)	DBP (mean ± SD)		
**Bernabe-Ortiz– 2022**	Peru	General population	26023	2500	-	44.8	-	-	≥140/90	23.5%
**Segura Vega– 2021**	Peru	General population	6253	1834	41.8	48.7	-	-	≥140/90	17.8%
**Muñoz– 2021**	Chile	General population (mine workers)	336	3000	36.2 ± 5.8	-	123.6 ± 12.4	80.9 ± 9.4	≥140/90	21.7%
**Chambergo-Michilot– 2021**	Peru	General population	12241	1500	-	-	-	-	≥ 130/80	50.5%
**Galdeano– 2021**	Argentina	General population	202	1800–4000	48.0 ± 1.4	38.1	124.0 ± 1.0	77.0 ± 1.0	≥140/90	23.8%
**Diaz-Lazo– 2021**	Peru	Hospitalized patients (patients with echocardiography studies)	488	3200	63.3 ± 17.7	42.6	117.2 ± 18.1	73.1 ± 12.4	≥140/90 or antihypertensive medication intake	28.5%
**Pérez-Galarza– 2021**	Ecuador	General population	4127	1500	51.5 ± 10.0	-	-	-	≥ 130/85	17.7%
**Menecier y Lomaglio– 2021**	Argentina	General population (only women)	105	3000	31.0 ± 8.9	42.6	115.5 ± 13.5	69.7 ± 10.3	≥ 130/85	16.2%
**Seclén– 2020**	Peru	General population	32950	3000	-	-	-	-	≥140/90	19.0%
**Bilo– 2020**	Peru	General population	289	4340	38.3 ± 13.2	50.5	111.3 ± 15.8	72.7 ± 10.1	≥140/90	6.9%
**Felix– 2020**	Ecuador	General population	2020	2800	51.4 ± 9.8	27.8	-	-	≥140/90 or antihypertensive medication intake	27.3%
**Sosa-Villarreal– 2020**	Bolivia	Hospitalized patients (with chronic cor pulmonale)	162	3577–4050	68.7 ± 12	47.5	-	-	≥130/80	39.0%
**Mamani-Ortiz– 2019**	Bolivia	General population	10704	3500	37.8 ± 18	42.6	-	-	≥130/85 or antihypertensive medication intake	17.2%
**Nieto-Martínez– 2018**	Venezuela	General population	412	1600–3000	44.1 ± 1.4	-	119.9 ± 1.4	75.1 ± 1.4	≥140/90 or antihypertensive medication intake	31.3%
**Bernabé-Ortiz– 2017**	Peru	General population	1155	3825	-	68.3	-	-	≥140/90	31.0%
**Hernández-Hernández– 2017**	Mexico	General population	134	3241–2981	44.2 ± 14.5	22.3	-	-	140/90 or antihypertensive medication intake	37.3%
**Ninatanta-Ortiz– 2016**	Peru	General population (only students and mothers)	841	2388–2750	29.2 ± 6.7	18.3	-	-	≥130/85	6.0%
**Burroughs Peña– 2015**	Peru	General population	1004	3825	55.3 ± 12.4	48.3	-	-	≥140/90	11.0%
**Ojeda– 2014**	Peru	General population (only women)	771	2577–3570	48.4 ± 4.9	-	-	-	≥140/90 or antihypertensive medication intake	6.0%
**Medina-Lezama– 2007**	Peru	General population	1878	2335	49 ± 17.2	46.1	109.3 ± 14.1	72.9 ± 9.1	≥140/90	16.0%
**De Guimaraes– 2007**	Peru	General population	204	3100	43.3	37.7	-	-	≥140/90	11.2%
**Baracco– 2007**	Peru	General population	99	4100	47.4 ± 12.9	48.9	111.0 ± 16.0	72.2 ± 9.3	≥130/85 or antihypertensive medication intake	18.1%
**Bernabé-Ortiz– 2017**	Peru	General population (Poor peri urban communities)	201	2761	-	47.2	120.9 ± 18.7	74.2 ± 9.2	≥140/90 or antihypertensive medication intake	12.0%
**Sempértegui– 2010**	Ecuador	General population (only elderly people)	352	2800	74.5 ± 6.2	36.0	-	-	≥130/80	51.1%
**Pajuelo– 2012**	Peru	General population	959	3000	-	49.9	109.3 ± 15.5	63.7 ± 11.3	≥130/85	15.0%
**Camacho– 2016**	Colombia	General population	2085	2000	-	-	-	-	≥140/90	36.0%
**Armaza Cespedes– 2016**	Bolivia	General population (only military people)	204	2558	-	85.2	119.10 ± 76.13	69.7 ± 10.3	≥130/85	6.0%
**Hernández-Vásquez– 2019**	Peru	General population	10874	2000	-	-	-	-	≥130/80	29.4%
**Hernández-Vásquez– 2019**	Peru	General population	10874	2000	-	-	-	-	≥140/90	11.0%
**Díaz Lazo– 2006**	Peru	Hospitalized patients	137	3250	60.5 ± 10.8	39.4	135.3 ± 27.3	83.6 ± 13.3	≥140/90 or antihypertensive medication intake	39.4%
**Santos– 2001**	Chile	General population (people of an aymara ethnic group)	196	2350, 5390	47.4 ± 18.6	39.8	122.2 ± 17.5	71.9 ± 10.5	≥140/90 or antihypertensive medication intake	18.4%

m.a.s.l.: meters above sea level, SBP: systolic blood pressure, DBP: diastolic blood pressure, SD: standard deviation, (—): not reported.

### Statistical analyses

We performed the analysis with the STATA V16.0 program. We performed meta-analyses according to the cut-off point for hypertension (≥140/90 mmHg, ≥130/85 mmHg, ≥ 130/80 mmHg). For this purpose, we calculated the pooled prevalences using a random-effects model, with their 95% confidence intervals calculated using the exact method. We used the Freeman-Tukey Double Arcsine transformation to stabilize variances. To assess heterogeneity and its sources, we used the I^2^ test and performed subgroup analyses according to population: sex, country, risk of bias, and altitude. In addition, we meta-analyzed the prevalence of the studies in the hospital population. We decided not to use Egger’s test or funnel plots, as their usefulness for assessing publication bias in the meta-analysis of prevalence is unclear and the results may reflect selection bias.

As a post hoc analysis, we performed a meta-regression analysis to evaluate the effect of variables such as the mean age of the participants, altitude, the year in which the study was published and the risk of bias score. In the case of altitude, we used the mean altitude reported by the study. If only the minimum altitude was reported, we used that value to perform the analysis.

## Results

### Selection of studies

**Fig 1:**
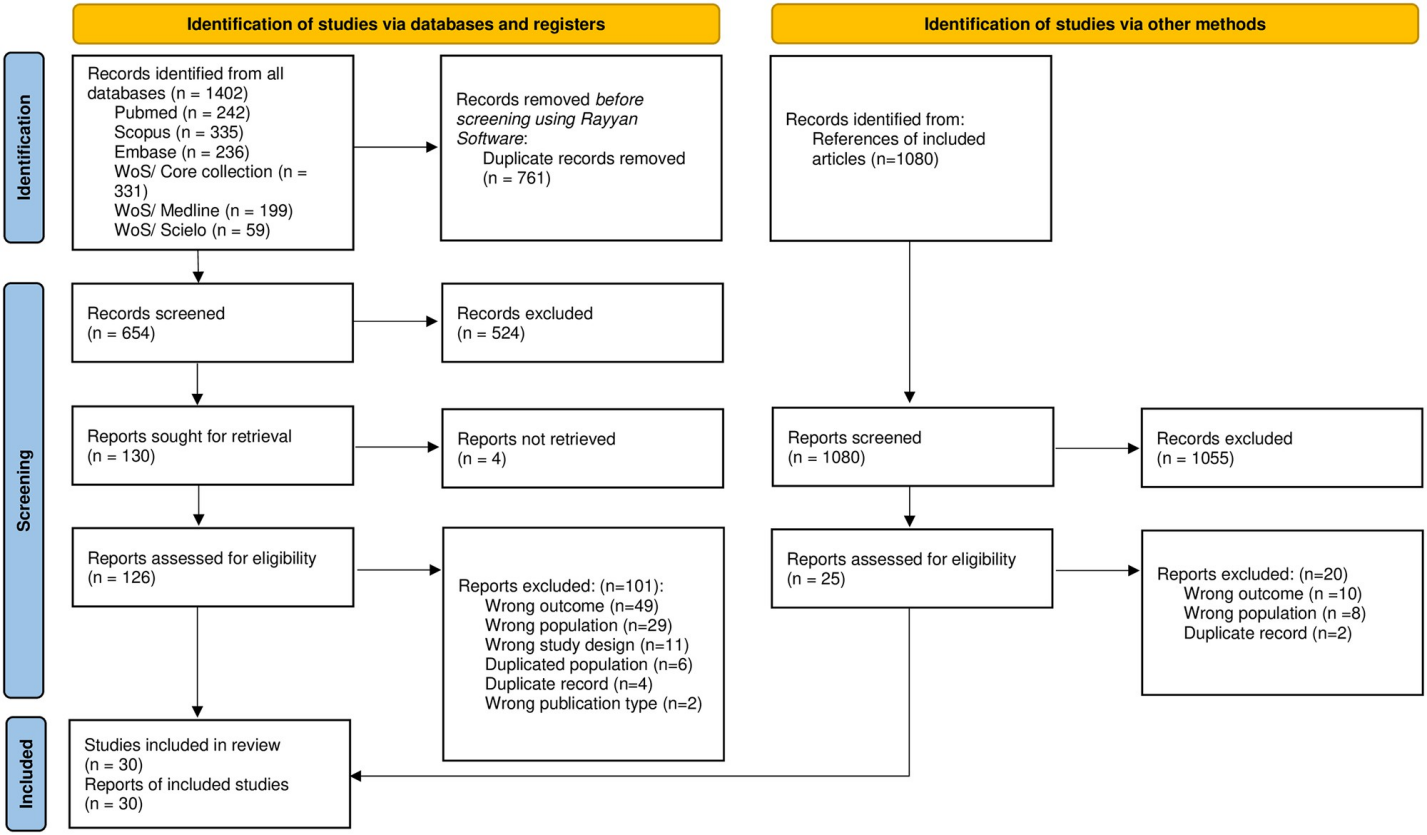
Flow diagram summarizing the process of literature search and selection. N: number of studies.

After removing duplicates, 654 studies were evaluated by title and abstract. Of these, 130 studies were reviewed in full-text. Ultimately, 30 studies were included in our analysis, with 25 sourced from the database search and 5 from the reference review of the included studies (Fig 1). The full-text studies that were excluded and the reasons for their exclusion can be found in S3 Table.

### Characteristics of the included studies

All studies that met the eligibility criteria were cross-sectional studies [27–56] (Table 1). The number of participants in all studies was 117 406 and ranged from 99 to 32 950 for the individual studies. The mean age ranged from 29.2 to 74.5 years. The altitude level of residence of the study participants ranged from 1 500 to 5 390 m.a.s.l. The countries where the studies were carried out were: Peru, [29–31, 36, 38–44, 46, 49, 50, 52, 53, 55] Ecuador [57–59] Bolivia [60–62] Argentina [28, 56], Chile [51, 54], Colombia [63], Venezuela [64], and Mexico [65]. Twenty-seven studies were carried out in the general population [27, 28, 30–32, 34–49, 51–56], and three in hospitals [29, 33, 50]. Some studies evaluated the prevalence of hypertension in specific populations, such as: women [66, 67]; older adults (65 years and older) [59]; patients with chronic cor pulmonale [62]; high school students, university students and mothers of elementary school students [68]; air force military [60]; rural, urban, and rural-to-urban migrant populations [69]; and patients who have echocardiographic studies [29]. On the other hand, only 2 studies specified that their populations were comprised of individuals who were "natives," meaning they were born and raised in the area where they currently reside [70, 71]. Additionally, only 3 studies specified that their populations were "altitude residents," defined as individuals who were not born at high altitude, but have lived there for more than 12 months [61, 66, 72].

Different blood pressure cut-off points were used for the definition of arterial hypertension. Twenty studies defined it as a blood pressure of ≥140/90 mmHg [29–32, 35–37, 39–42, 44, 47, 49–54, 56], 7 studies as ≥130/85 mmHg [57, 60, 61, 67, 68, 73, 74]; and 4 studies as ≥130/80 mmHg [36, 45, 49, 55]. In addition, 11 studies took into account the use of antihypertensive drugs for the definition of hypertension [29, 32, 34–36, 40, 43, 51, 75].

### Prevalence of hypertension in residing at altitude in Latin America and the Caribbean

When performing meta-analyses to assess the prevalence of hypertension in the general population, we found that out of 17 studies [30–32, 35, 36, 39–42, 44, 47, 49, 51–54, 56] that used the cut-off point ≥ 140/90 mmHg the prevalence of hypertension was 19.1% (95% CI: 16.1 to 22.2; I^2^: 99.0%) with a range of 6.1 to 37.3%. In the 7 studies [57, 60, 61, 67, 68, 73, 74] that used the cut-off point of ≥ 130/85 mmHg was 13.1% (95% CI: 9.9 to 16.6; I^2^: 95.1%) with a range of 5.6 to 17.7%. The prevalence of hypertension in 3 studies [33, 45, 55] using the cut-off point of ≥ 130/80 mmHg was 43.4% (95% CI: 27.1 to 60.5) (Table 2).

**Table 2:** Overall and subgroups prevalence of hypertension in the general population of the altitude.

	≥ 140/90 mmHg			≥ 130/85 mmHg			≥ 130/80 mmHg		
	n studies	Prevalence (95% CI)	I^2^ (%)	n studies	Prevalence (95% CI)	I^2^ (%)	n studies	Prevalence (95% CI)	I^2^ (%)
**Overall**	18	19.1 (16.1 to 22.2)	99.0	7	13.1 (9.9 to 16.6)	95.7	3	43.4 (27.1 to 60.5)	NE
**Sex**									
Male	5	22.3 (12.3 to 34.3)	96.1	5	13.5 (7.6 to 20.8)	93.8	1	55.1 (46.4 to 63.7)	NE
Female	6	15.1 (8.3 to 23.5)	97.4	6	11.7 (7.4 to 16.7)	91.9	1	48.9 (42.2 to 55.6)	NE
**Country**									
Peru	11	14.5 (11.5 to 17.8)	99.2	3	12.0 (5.0 to 21.5)	NE	2	40.3 (39.7 to 41.0)	NE
Ecuador	1	27.3 (25.4 to 29.3)	NE	1	17.7 (16.5 to 18.9)	NE	1	51.1 (45.8 to 56.5)	NE
Chile	2	20.5 (17.1 to 24.0)	NE						
Venezuela	1	31.3 (26.9 to 36.0)	NE						
Mexico	1	37.3 (29.1 to 46.1)	NE						
Colombia	1	36.0 (34.0 to 38.1)	NE						
Argentina	1	23.8 (18.1 to 30.2)	NE	1	16.2 (9.7 to 24.7)	NE			
Bolivia				2	16.9 (16.2 to 17.6)	NE			
**Risk of bias**									
Low risk	7	22.7 (16.3 to 30.0)	99.5	1	17.3 (16.6 to 18.0)	NE	2	40.3 (39.7 to 41.0)	NE
Moderate risk	11	16.8 (13.0 to 21.0)	98.3	5	13.8 (8.5 to 20.2)	96.2			
High risk				1	5.9 (3.1 to 10.0)	NE	1	51.1 (45.8 to 56.5)	NE
**Altitude (m.a.s.l)**									
Intermediate (1500 to 2500)	7	21.3 (14.5 to 29.0)	99.3	2	15.2 (14.2 to 16.2)	NE	2	40.3 (39.7 to 41.0)	NE
High (2500 to 3500)	8	18.7 (15.6 to 22.1)	98.0034	3	11.9 (6.1 to 19.3)	NE	1	51.1 (45.8 to 56.5)	NE
Very high (>3500)	3	15.0 (3.8 to 31.7)	NE	2	17.1 (16.4 to 17.8)	NE			

95% CI: 95% confidence interval, NE: not evaluated, m.a.s.l: meters above the sea level.

### Subgroup analysis

The prevalence of hypertension in the general population, measured with different blood pressure cut-off points, was evaluated according to sex, country, risk of bias, and altitude (Table 2).

With respect to sex, the point estimate of the prevalence of hypertension as ≥ 140/90 mmHg was higher in men (22.3%; 95% CI: 12.3 to 34.3; I2: 96.1%) than in women (15.1%; 95% CI: 8.3 to 23.5; I^2^: 97.4%); as ≥ 130/85 mmHg, it was higher in men (13.5%; 95% CI: 7.6 to 20.8; I^2^: 93.8%) than in women (11.7%; 95% CI: 7.4 to 16.7; I^2^: 91.1%); and as ≥ 130/80 mmHg was also higher in men (55.1%; 95% CI: 46.4 to 63.7) than in women (48.9%; 95% CI: 42.2 to 55.6). However, the confidence intervals overlap so it is not possible to extrapolate this trend to the population of interest.

Regarding the country, the prevalence of hypertension with cut-off point ≥ 140/90 mmHg, was higher in Mexico (37.3%; 95% CI: 29.1 to 46.1), while it was lower in Peru (14.5%; 95% CI: 11.5 to 17.8; I^2^: 99.2%); with cut-off point ≥ 130/85 mmHg, it was higher in Ecuador (17.7%; 95% CI: 16.5 to 18.9) and lower in Peru (12.0%; 95% CI: 5.0 to 21.5); and with cut-off point ≥ 130/80 mmHg, prevalence was also higher in Ecuador (51.1%; 95% CI: 45.8 to 56.5) and lower in Peru (40.3%; 95% CI: 39.7 to 41.0).

When we evaluated the prevalences according to risk of bias, we found that studies with moderate and low risk of bias showed similar results for the ≥140/90 mmHg cut-off point. On the other hand, for the ≥130/85 mmHg cut-off point, studies with lower risk of bias showed higher point prevalences and studies with higher risk of bias showed lower prevalences. For the ≥130/80 mmHg cut-off point the high risk of bias study showed a higher prevalence than the low-risk studies.

Regarding altitude levels, prevalences were similar among studies conducted at intermediate, high and very high altitudes.

### Meta-regression analyses

In the meta-regression analyses, no association was found between the altitude (p = 0.256), mean age of the participants (p = 0.921), the year of publication (p = 0.432) and risk of bias score (p = 0.206) with the prevalence of hypertension with the cut-off point of ≥140/90 (S1 Fig).

### In-hospital prevalence of hypertension

The in-hospital prevalence of hypertension was observed in 2 studies using the cut-off point of ≥ 140/90 mmHg and 1 study using a cut-off point of ≥ 130/80 mmHg, which showed a prevalence of 31.0% (95% CI: 25.9 to 36.4) and 38.9% (95% CI: 31.3 to 46.9), respectively.

### Risk of bias

When evaluating the risk of bias, more than 60% of the studies met criteria for adequate sample size, detailed description of subjects and setting, appropriate sampling frame and methods, and reliable measurement of conditions in all participants. Half of the studies met criteria for appropriate statistical analysis. However, only 14 studies utilized validated methods for identifying hypertension, including consideration of a history of treatment with antihypertensive drugs. Without this criterion, the prevalence of hypertension in these studies may be underestimated. Additionally, only 13 studies met criteria for data analysis with sufficient coverage of the identified sample, which could potentially lead to underestimation or overestimation of the prevalence, depending on factors such as the sex and age of the participants. The overall assessment score can be found in Table 1 and more detailed information can be found in S4 Table.

## Discussion

### Main findings

In this systematic review, we found that the prevalence of hypertension at altitude in Latin America and the Caribbean, was 19.1%, according to the cut-off point of ≥140/90 mmHg; 13.1% according to the cut-off point of ≥130/85 mmHg and 43.4% according to the cut-off point of ≥ 130/80 mmHg. Furthermore, there was a tendency for the prevalence of hypertension to be higher in men. Meta-regression analyses showed no association between the prevalence of hypertension and altitude, age, and year of publication.

The prevalence of hypertension (≥ 140/90 mmHg) found in the present review at altitude in Latin America and the Caribbean is lower than that found in altitude and non-altitude populations globally (32.3%) and in Latin America and the Caribbean (39.1%) [76]. This could be partially explained by the larger population in rural areas at altitude, where the prevalence of hypertension has been shown to be lower [77]. Lower pollution and greater exposure to green spaces could also contribute to this result [78, 79]. In addition, other factors such as lifestyle and diet could explain this finding [80]. However, information in this population is scarce.

Regarding the prevalence of hypertension in altitude populations, a systematic review that evaluated the prevalence in Tibetan and non-Tibetan residents at an altitude of over 3000 m.a.s.l. found prevalences ranging from 15.2 to 71.8% [81]. In addition, another systematic review in Tibetan population found prevalences between 23 to 56% [82]. It can be seen that the prevalence ranges are higher compared to the present review where the prevalences were between 10.6 to 37.3% for the cut-off point of 140/90 mmHg. However, none of these reviews meta-analyzes the prevalences to find a global estimate.

In addition, these reviews performed meta-regression analyses to assess the association between altitude and blood pressure. One review found no significant difference between altitude and diastolic and systolic blood pressure [11]. The another systematic review found that for every 100 meters of altitude the prevalence of hypertension increased by 2% with a close statistical significance (p = 0.06) [13]. These findings are in agreement with the present review, where we found no statistically significant association between altitude and the prevalence of hypertension. However, these results are imprecise and should be corroborated in future reviews with a larger number of studies.

The tendency to a higher prevalence of hypertension in the Tibetan population could be due to the physiological changes, such as improved levels of hypoxic ventilation response, blood saturation, pulmonary function, maximal cardiac output, lower levels of pulmonary vasoconstriction, and higher hemoglobin concentrations [15, 83]. In addition, sodium-rich diet, high alcohol intake, genetic modification of the NOS3 (Nitric Oxide Synthase 3) gene and ADD1 (Adducin 1), could explain the high prevalence of hypertension in this same population as they are risk factors [84, 85].

On the contrary, the lower prevalence of hypertension in the altitude population of Latin America and the Caribbean could be attributed to the fact that hypoxia—hypobaric [86] leads to smooth muscle relaxation, increased collateral circulation and vascularization [87]. In particular, the Andean population characterized by lower risk factors such as low socio-cultural status, increased mineral intake, healthier diets, larger pulmonary residual volume, smaller aortic thickness and lower cardiac output [80]. Furthermore, Andean population experiences lower rates of obesity, and diabetes [88–90]. This can be partially attributed to the influence of high altitude on reduced energy intake and increased resting metabolic rate [91]. Also, this population engages in higher levels of physical activity [92, 93], likely driven by demanding agricultural and livestock tasks. These factors could explain why altitude inhabitants of Latin America and the Caribbean have lower blood pressure.

### Subgroup findings

In the general population at altitude, there was a tendency for the prevalence of hypertension to be higher in men, which is consistent with the systematic review by Nirmal et al. where they found an increase in systolic and diastolic blood pressure at higher altitudes in men [11]. Lower prevalence in women could be explained by the hormonal production of estradiol that generates a vasodilator effect by increasing endothelial nitric oxide synthase synthesis and activation of K+ channels [94, 95].

According to country, Mexico has the highest prevalence of hypertension (37.3%, ≥140/90 mmHg) although this is not consistent with that found by Hernandez et al. who evidence a lower prevalence of hypertension in the general population of Mexico City (11.7%) [96]. In contrast, the lowest prevalence was found in Peru (14.5%, ≥140/90 mmHg) according to 11 studies, lower than that found in the systematic review by Andrea et al. who evaluated the prevalence of hypertension (≥140/90 mmHg) in Peru including 15 studies and finding a point prevalence of 22.0% (95% CI: 20.0% to 25.0%). Possibly this is because they included studies with a scope of sea level populations with the presence of urban areas and populations with a high prevalence of factors associated with hypertension such as obesity and type 2 diabetes [97]. These findings can be used to guide tailored health interventions in specific geographic contexts. In addition, exploring the underlying mechanisms responsible for the reduced hypertension prevalence in this altitude range could uncover novel insights into the interplay of altitude, environmental factors, and genetic predispositions in shaping cardiovascular health.

### Limitations of included studies

Of all the included studies, 58% did not present data analysis with sufficient coverage of the identified sample, 48% did not have an appropriate statistical analysis, and 55% did not assess antihypertensive treatment within their definition of hypertension. In addition, a large proportion of the studies did not present data on the type of resident; therefore, a subgroup analysis could not be performed with the categories of: resident <12 months, resident >12 months, and native. In the studies that used various altitude level classification groups, an approximate value of such data was placed. However, most of them were conducted in the general population and with large populations.

### Implications of the results for public health policies and recommendations for future studies

In the present review, we found a lower prevalence of hypertension in the altitude population of Latin America and the Caribbean compared to other altitude and non-altitude populations. However, it is important to acknowledge that regions situated at elevated altitudes exhibit certain vulnerabilities, encompassing inadequacies in essential primary healthcare services, restricted and uneven access to essential medications, and a significant lack of effective programs for the prevention, treatment, and management of hypertension [98–100]. As a result, complications of the disease may present earlier. To address these challenges, recommendations include strengthening primary healthcare through standardized protocols, implementing progress evaluation systems, fostering a supportive community-healthcare-patient relationship, and applying a comprehensive intervention driven by the collaboration of healthcare providers, and the community [101–103]. More studies are needed to evaluate the factors that lead to a lower prevalence of hypertension at altitude, such as length of residence, ethnic origin, in other countries, and other particular characteristics of these populations, such as environmental and lifestyle factors [104].

### Limitations and strengths of the review

This is the first systematic review to synthesize information on the prevalence of hypertension in adults living at altitude in Latin America and the Caribbean. An exhaustive search strategy was employed and in local databases such as Scielo, in addition, 3 cut-off points for the definition of hypertension (≥140/90 mmHg, ≥130/85 mmHg and ≥130/80 mmHg) were considered. However, we recognize some limitations; the systematic search in gray literature was not performed. Moreover, our findings reveal imprecision in the prevalence results, exemplified by the lower point prevalence hypertension of the 140/90 cut-off point compared to the 130/85 cut-off point. Likewise, meta-regression analyses should be interpreted with caution due to the lack of statistical power resulting from the small number of studies included.

## Conclusion

The prevalence of hypertension in altitude populations in Latin America and the Caribbean was 19.1% (≥ 140/90 mm Hg), 13.1% (≥ 130/85 mm Hg) and 43.4% (≥ 130/80 mm Hg). It is higher in men and in countries in Mexico and Ecuador and lower in Peru. The results of this systematic review suggest that the prevalence of hypertension in populations living at altitude in Latin America and the Caribbean is lower than that reported in populations living at sea level and lower than in other altitude populations such as Tibetans. We recommend that these differences be taken into account for strategies and interventions in clinical practice, public policies and future research in these populations that have particular characteristics in their environment and lifestyle related to hypertension.

## Supporting information

S1 Fig Meta-regression analyses. (DOCX)

S1 Table PRISMA checklist 2020. (DOCX)

S2 Table Search strategy. (DOCX)

S3 Table Excluded studies and reasons. (DOCX)

S4 Table Risk of bias of included studies using the Joanna Briggs Institute Critical Appraisal Tool for prevalence studies. (DOCX)
