# Supplementary material for: Prevalence of hypertension in adults living at altitude in Latin America and the Caribbean: A systematic review and meta-analysis
Source: PLoS One. 2023 Oct 12;18(10):e0292111. doi: 10.1371/journal.pone.0292111 (PMC10569637; doi:10.1371/journal.pone.0292111)
Supplement: S1 Fig — (DOCX) [file pone.0292111.s001.docx]

a. b.


c. d.

**Supplementary material 4**. Meta-regression analyses. a. LogOdds of hypertension (≥140/90) versus altitude (m.a.s.l) (p=0.256), b. LogOdds of hypertension (≥140/90) versus mean age (years) (p=0.921), c. LogOdds of hypertension (≥140/90) versus year of publication (p=0.432), d. LogOdds of hypertension (≥140/90) versus risk of bias score (p=0.324).
