# Supplementary material for: Prevalence of hypertension in adults living at altitude in Latin America and the Caribbean: A systematic review and meta-analysis
Source: PLoS One. 2023 Oct 12;18(10):e0292111. doi: 10.1371/journal.pone.0292111 (PMC10569637; doi:10.1371/journal.pone.0292111)
Supplement: S2 Table — (DOCX) [file pone.0292111.s003.docx]

## Supplementary material 2. Search strategy

**Search date:** since 2000 to January 10, 2023

| Database | Search strategy | Search |
| --- | --- | --- |
| PubMed | **#1 Hypertension**  “Hypertension”[MeSH] OR “Hypertensi*”[TIAB] OR (“Blood Pressure*”[TIAB] AND “High”[TIAB]) OR (“Blood Pressure*”[OT] AND “High”[OT]) OR “Hypertensi*”[OT]  **#2 Altitude**  “Altitude”[MeSH] OR “Altitude*”[TIAB] OR “mountain*”[TIAB] OR “highland*”[TIAB] OR “Andes”[TIAB] OR “Andean*”[TIAB] OR “Altitude*”[OT] OR “mountain*”[OT] OR “highland*”[OT] OR “Andes”[OT] OR “Andean*”[OT]  **#3 Latin America**  Latin America[Mh] OR "Latin America"[tiab] OR Caribbean Region[Mh] OR "Caribbean Region"[tiab] OR South America [Mh] OR "South America”[tiab] OR Indians, South American [Mh] OR Hispanoamerica*[tiab] OR Iberoamerica*[tiab] OR Panamerican*[tiab] OR Argentina[Mh] OR Argentina[tiab] OR Argentin*[ad] OR Bolivia[Mh] OR Bolivia[tiab] OR Bolivia[ad] OR Brazil[Mh] OR Brazil[tiab] OR Brazil*[ad] OR Brasil*[ad] OR Chile[Mh] OR Chile[tiab] OR Colombia[Mh] OR Colombia[tiab] OR Colombia[ad] OR Costa rica[Mh] OR Costa rica[tiab] OR Costa Ric*[ad] OR Cuba[Mh] OR Cuba[tiab] OR Ecuador[Mh] OR Ecuador[tiab] OR Ecuador*[ad] OR El salvador[Mh] OR "El salvador"[tiab] OR "El salvador"[ad] OR Guatemala[Mh] OR Guatemala[tiab] OR Guatemala[ad] OR Haiti[Mh] OR Haiti[tiab] OR Honduras[Mh] OR Honduras[tiab] OR Mexico[Mh] OR Mexico[tiab] OR Mexico[ad] OR Mejico[ad] OR Nicaragua[Mh] OR Nicaragua[tiab] OR Panama[Mh] OR Panama[tiab] OR Paraguay[Mh] OR Paraguay[tiab] OR Paraguay[ad] OR Peru[Mh] OR Peru[tiab] OR Peru*[ad] OR Puerto Rico[Mh] OR "Puerto Rico"[tiab] OR "Puerto Rico"[ad] OR Dominican Republic[Mh] OR "Dominican Republic"[tiab] OR "Dominican Republic"[ad] OR Uruguay[Mh] OR Uruguay[tiab] OR Uruguay[ad] OR Venezuela [Mh] OR Venezuela [tiab] OR Venezuela [ad] OR Suriname[Mh] OR Suriname[tiab] OR Surinam*[ad] OR Guiana*[tiab] OR Guiana*[ad] OR Guyan*[tiab] OR Guyan*[ad]  #1 AND #2 AND #3 | 242 |
| Scopus | **#1 Hypertension**  TITLE-ABS-KEY(“Hypertensi*”) OR TITLE-ABS-KEY(“Blood Pressure*” W/2 “High”)  **#2 Altitude**  TITLE-ABS-KEY(“Altitude*” OR “mountain*” OR “highland*” OR “Andes” OR “Andean*”)  **#3 Latin America**  AFFILCOUNTRY ( argentina OR bolivia OR brazil OR brasil OR colombia OR chile OR ecuador OR guyana OR "french Guiana" OR paraguay OR peru OR suriname OR uruguay OR venezuela OR belize OR "costa rica" OR "el Salvador" OR guatemala OR honduras OR nicaragua OR panama OR mexico OR mejico OR cuba OR "dominican republic" OR haiti OR jamaica OR "Puerto rico" OR "trinidad and tobago" OR barbados OR guadeloupe OR grenada OR martinique OR bermuda OR bahamas )  #1 AND #2 AND #3 | 335 |
| WoS | **#1 Hypertension**  TS=(“Hypertensi*”) OR TS=(“Blood Pressure*” NEAR/2 “High”)  **#2 Altitude**  TS=(“Altitude*” OR “mountain*” OR “highland*” OR “Andes” OR “Andean*”)  **#3 Latin America**  CU= (argentina OR bolivia OR brazil OR brasil OR colombia OR chile OR ecuador OR guyana OR "french Guiana" OR paraguay OR peru OR suriname OR uruguay OR venezuela OR belize OR "costa rica" OR "el Salvador" OR guatemala OR honduras OR nicaragua OR panama OR mexico OR mejico OR cuba OR "dominican republic" OR haiti OR jamaica OR "Puerto rico" OR "trinidad and tobago" OR barbados OR guadeloupe OR grenada OR martinique OR bermuda OR bahamas)  #1 AND #2 AND #3 | 331 |
| SciELO | **#1 Hypertension**  TS=(“Hypertensi*”) OR TS=(“Blood Pressure*” NEAR/2 “High”)  **#2 Altitude**  TS=(“Altitude*” OR “mountain*” OR “highland*” OR “Andes” OR “Andean*”)  **#3 Latin America**  CU= (argentina OR bolivia OR brazil OR BRASIL OR colombia OR chile OR ecuador OR guyana OR "french Guiana" OR paraguay OR peru OR suriname OR uruguay OR venezuela OR belize OR "costa rica" OR "el Salvador" OR guatemala OR honduras OR nicaragua OR panama OR mexico OR mejico OR cuba OR "dominican republic" OR haiti OR jamaica OR "Puerto rico" OR "trinidad and tobago" OR barbados OR guadeloupe OR grenada OR martinique OR bermuda OR bahamas) OR AD= (argentina OR bolivia OR brazil OR BRASIL OR colombia OR chile OR ecuador OR guyana OR "french Guiana" OR paraguay OR peru OR suriname OR uruguay OR venezuela OR belize OR "costa rica" OR "el Salvador" OR guatemala OR honduras OR nicaragua OR panama OR mexico OR mejico OR cuba OR "dominican republic" OR haiti OR jamaica OR "Puerto rico" OR "trinidad and tobago" OR barbados OR guadeloupe OR grenada OR martinique OR bermuda OR bahamas)  #1 AND #2 AND #3 | 59 |
| Medline | **#1 Hypertension**  MH=(Hypertension) OR TS=(“Hypertensi*”) OR TS=(“Blood Pressure*” NEAR/2 “High”)  **#2 Altitude**  MH=(“Altitude”) OR TS=(“Altitude*” OR “mountain*” OR “highland*” OR “Andes” OR “Andean*”)  **#3 Latin America**  AD= (argentina OR bolivia OR brazil OR BRASIL OR colombia OR chile OR ecuador OR guyana OR "french Guiana" OR paraguay OR peru OR suriname OR uruguay OR venezuela OR belize OR "costa rica" OR "el Salvador" OR guatemala OR honduras OR nicaragua OR panama OR mexico OR mejico OR cuba OR "dominican republic" OR haiti OR jamaica OR "Puerto rico" OR "trinidad and tobago" OR barbados OR guadeloupe OR grenada OR martinique OR bermuda OR bahamas)  #1 AND #2 AND #3 | 199 |
| Embase | **#1 Hypertension**  Hypertension/exp OR (‘Hypertensi*’):ti,ab,kw OR (‘Blood Pressure*’ NEAR/2 ‘High’):ti,ab,kw  **#2 Altitude**  Altitude/exp OR (‘Altitude*’ OR ‘mountain*’ OR ‘highland*’ OR ‘Andes’ OR ‘Andean*’):ti,ab,kw  **#3 Latin America**  'argentina':ca OR 'bolivia':ca OR 'brazil':ca OR brasil:ca OR 'colombia':ca OR 'chile':ca OR 'ecuador':ca OR 'guyana':ca OR 'french guiana':ca OR 'paraguay':ca OR 'peru':ca OR 'suriname':ca OR 'uruguay':ca OR 'venezuela':ca OR 'belize':ca OR 'costa rica':ca OR 'el salvador':ca OR 'guatemala':ca OR 'honduras':ca OR 'nicaragua':ca OR 'panama':ca OR 'mexico':ca OR mejico:ca OR 'cuba':ca OR 'dominican republic':ca OR 'haiti':ca OR 'jamaica':ca OR 'puerto rico':ca OR 'trinidad and tobago':ca OR 'barbados':ca OR 'guadeloupe':ca OR 'grenada':ca OR 'martinique':ca OR 'bermuda':ca OR 'bahamas':ca  #1 AND #2 AND #3 | 236 |
