# Supplementary material for: Prevalence of hypertension in adults living at altitude in Latin America and the Caribbean: A systematic review and meta-analysis
Source: PLoS One. 2023 Oct 12;18(10):e0292111. doi: 10.1371/journal.pone.0292111 (PMC10569637; doi:10.1371/journal.pone.0292111)
Supplement: S3 Table — (DOCX) [file pone.0292111.s004.docx]

**Supplementary material 3.** **Excluded studies and reasons**

| N° | Author, year | Title | Reason for exclusion |
| --- | --- | --- | --- |
| 1 | Lazo-2021 | Factors associated with left ventricular hypertrophy in adults living at a high altitude city in peru. | Duplicate |
| 2 | Galdeano-2021 | Evaluation of cardiovascular variables in a calchaquã­ population in the middle and high mountains of tucumã¡n | Duplicate |
| 3 | Viamonte-2020 | Nutritional status in adults of rural population in a canton of the ecuadorian highlands | Duplicate |
| 4 | Sosa-villarreal-2020 | Cardiac arrhythmias in highlanders with a diagnosis of chronic cor pulmonale. | Duplicate |
| 5 | Bernabe-ortiz-2021 | Association between body mass index and blood pressure levels across socio-demographic groups and geographical settings: analysis of pooled data in peru. | Duplicate population |
| 6 | Miranda-2019 | Multimorbidity at sea level and high-altitude urban and rural settings: the cronicas cohort study. | Duplicate population |
| 7 | Pastorius-2010 | Normative values and correlates of carotid artery intima-media thickness and carotid atherosclerosis in andean-hispanics: the prevencion study. | Duplicate population |
| 8 | Nishimura-2020 | Individual variations and sex differences in hemodynamics with percutaneous arterial oxygen saturation (spo_2_) in young andean highlanders in bolivia. | Wrong outcome |
| 9 | Corante-2018 | Excessive erythrocytosis and cardiovascular risk in andean highlanders. | Wrong outcome |
| 10 | Kephart-2020 | Indoor air pollution concentrations and cardiometabolic health across four diverse settings in peru: a cross-sectional study. | Wrong outcome |
| 11 | Melby-2017 | Nutrition and physical activity transitions in the ecuadorian andes: differences among urban and rural-dwelling women. | Wrong outcome |
| 12 | Hernandez-vasquez-2020 | Depression in the peruvian population and its associated factors: analysis of a national health survey. | Wrong outcome |
| 13 | Salazar-lugo-2016 | Biochemical and nutritional factors associated with blood viscosity in adults living in a mountain chain, (imbabura), ecuador | Wrong outcome |
| 14 | Romero-2020 | Nutritional status in adults of rural population in a canton of the ecuadorian highlands | Wrong outcome |
| 15 | Galdeano-2021 | Evaluation of cardiovascular variables in a calchaquã­ population in the middle and high mountains of tucumã¡n | Wrong outcome |
| 16 | Lopez-pascual-2018 | Inverse association between metabolic syndrome and altitude: a cross-sectional study in an adult population of ecuador. | Wrong outcome |
| 17 | Galindo-2021 | Characteristics and clinical course of adult in patients with sars-cov-2 pneumonia at high altitude. | Wrong outcome |
| 18 | Estela-ayamamani-2015 | [physical performance of older adults living in rural areas at sea level and at high altitude in peru]. | Wrong outcome |
| 19 | Caravedo-2014 | Lack of association between chronic exposure to biomass fuel smoke and markers of right ventricular pressure overload at high altitude. | Wrong outcome |
| 20 | Oliveros-2021 | Basan index (body mass index, age, sex, arterial hypertension and neck circumference) predicts severe apnoea in adults living at high altitude. | Wrong outcome |
| 21 | Gulli-2007 | Autonomic regulation during orthostatic stress in highlanders: comparison with sea-level residents. | Wrong outcome |
| 22 | Lindgarde-2004 | Body adiposity, insulin, and leptin in subgroups of peruvian amerindians. | Wrong outcome |
| 23 | Gonzales-2013 | Association of high altitude-induced hypoxemia to lipid profile and glycemia in men and women living at 4,100m in the peruvian central andes. | Wrong outcome |
| 24 | Thomson-2021 | Potential protective effect from covid-19 conferred by altitude: a longitudinal analysis in peru during full lockdown. | Wrong outcome |
| 25 | Herrera-enriquez-2017 | Discordance of metabolic syndrome and abdominal obesity prevalence according to different criteria in andean highlanders: a community-based study. | Wrong outcome |
| 26 | Flood-2018 | Screening for chronic kidney disease in a community-based diabetes cohort in rural guatemala: a cross-sectional study. | Wrong outcome |
| 27 | Orces-2017 | The prevalence of metabolic syndrome among older adults in ecuador: results of the sabe survey. | Wrong outcome |
| 28 | Botero-2000 | Efficacy and safety of valsartan compared with enalapril at different altitudes. | Wrong outcome |
| 29 | Chirinos-2014 | Metabolic syndrome as an underlying disease entity and its relationship to subclinical atherosclerosis in andean hispanics. | Wrong outcome |
| 30 | Santos-martinez-2021 | [deterioration of gas exchange in subjects with an increase in body mass index at an altitude of 2,240 meters above sea level]. | Wrong outcome |
| 31 | Yovera-aldana-2021 | Nationwide prevalence and clinical characteristics of inpatient diabetic foot complications: a peruvian multicenter study | Wrong outcome |
| 32 | Moya-salazar-2021 | Risk factors in rural andean population with covid-19: a retrospective cohort study | Wrong outcome |
| 33 | Gonzales-2015 | Increased levels of serum î-glutamyltransferase and uric acid on metabolic, hepatic and kidney parameters in subjects at high altitudes | Wrong outcome |
| 34 | Galindo-2021 | Characteristics and clinical course of adult in-patients with sars-cov-2 pneumonia in bogota, colombia | Wrong outcome |
| 35 | Bernabe-ortiz-2016 | Geographical variation in the progression of type 2 diabetes in peru: the cronicas cohort study | Wrong outcome |
| 36 | Collazos-2011 | Characterization of patients requiring therapeutic phlebotomy in a blood bank at 8530 ft amsl | Wrong outcome |
| 37 | Siques-2009 | Blood pressure responses in young adults first exposed to high altitude for 12 months at 3550 m | Wrong outcome |
| 38 | Miele-2018 | Environmental exposures and systemic hypertension are risk factors for decline in lung function. | Wrong outcome |
| 39 | Schwartz-2015 | Sleep disordered breathing in four resource-limited settings in peru: prevalence, risk factors, and association with chronic diseases. | Wrong outcome |
| 40 | Baldeón-2021 | Prevalence of metabolic syndrome and diabetes mellitus type-2 and their association with intake of dairy and legume in andean communities of ecuador | Wrong outcome |
| 41 | Vinueza-2020 | Blood pressure in andean adults living permanently at different altitudes. | Wrong population |
| 42 | Taco-vasquez-2019 | Association between blood viscosity and cardiovascular risk factors in patients with arterial hypertension in a high altitude setting. | Wrong population |
| 43 | Simpson-2021 | Global reach 2018: andean highlanders, chronic mountain sickness and the integrative regulation of resting blood pressure. | Wrong population |
| 44 | Rodrã­guez-morã¡n-2008 | Cardiovascular risk factors and acculturation in yaquis and tepehuanos indians from mexico. | Wrong population |
| 45 | Amaru-2020 | Treatment of secondary erythrocytosis at high altitude | Wrong population |
| 46 | Romero-2020 | Gender differences in factors associated with hypertension in peru: analysis of the national demographic and health survey 2017 | Wrong population |
| 47 | Gonzalez-rivas-2016 | High prevalence and poor control of hypertension in five venezuelan populations: the vemsols study. | Wrong population |
| 48 | Gonzalez-garcia-2021 | Comorbidities of patients with idiopathic pulmonary fibrosis in four latin american countries. Are there differences by country and altitude? | Wrong population |
| 49 | Zavala-loayza-2016 | Characteristics associated with antihypertensive treatment and blood pressure control: a population-based follow-up study in peru. | Wrong population |
| 50 | Grandi-2013 | Effect of high altitude on birth weight and adverse perinatal outcomes in two argentine populations. | Wrong population |
| 51 | Moyano-2021 | Prevalence of stroke survival in rural communities living in northern peru. | Wrong population |
| 52 | O'donnell-2011 | Prevalence of and risk factors for chronic kidney disease in rural nicaragua. | Wrong population |
| 53 | Guevara-2015 | Socioeconomic and lifestyle factors associated with chronic conditions among older adults in ecuador. | Wrong population |
| 54 | Dickstein-2014 | Epidemiologic profile of patients seen in primary care clinics in an urban and a rural setting in haiti, 2010-11. | Wrong population |
| 55 | Caravita-2016 | Role of acetazolamide and telmisartan/nifedipine-gits combination in antagonizing the blood pressure rise induced by high altitude exposure | Wrong population |
| 56 | Fandiño-2016 | People living at higher elevations have a predisposing risk factor to the choice of food high in carbohydrates: dm ii effects | Wrong population |
| 57 | Aquini-2007 | Yoga for the elderly: aiming for the top of the mountain | Wrong population |
| 58 | Bilo-2013 | Combined antihypertensive treatment and blood pressure responses to acute high altitude exposure in patients with hypertension. Highcare-andes lowlanders study | Wrong population |
| 59 | Rivero-2012 | Non alcoholic fatty liver disease in patients with metabolic syndrome: population study in the libertador municipality, merida state | Wrong population |
| 60 | Faini-2014 | Blood pressure recovery after maximal exercise at high altitude in mild hypertensive subjects and effects of antihypertensive combination treatment | Wrong population |
| 61 | Diaz-lazo-2021 | Clinical and epidemiological characteristics of patients with covid-19 at a high-altitude hospital | Wrong population |
| 62 | Giraldo-2020 | Gender differences in factors associated with hypertension in peru: analysis of the national demographic and health survey 2017. | Wrong population |
| 63 | Seclen-2017 | Elevated incidence rates of diabetes in peru: report from perudiab, a national urban population-based longitudinal study. | Wrong population |
| 64 | Mejia-2016 | Age as a risk factor for developing metabolic syndrome in mine workers at high altitude. | Wrong population |
| 65 | Eliana-2014 | It is ethnicity or living altitude what increments climacteric symptoms? A study in andean regions. | Wrong population |
| 66 | Chambergo-michilot-2021 | Socioeconomic determinants of hypertension and prehypertension in peru: evidence from the peruvian demographic and health survey. | Wrong population |
| 67 | Miranda-2013 | Major cardiovascular risk factors in latin america: a comparison with the united states. The latin american consortium of studies in obesity (laso) | Wrong population |
| 68 | Edward-2013 | Prevalencia del síndrome metabólico en una población adulta rural de los andes venezolanos: explorando diferentes criterios de diagnóstico y su grado de acuerdo | Wrong population |
| 69 | Jose-2018 | Health evaluation for occupational highaltitude exposure: results from a chilean copper mine during 2016 | Wrong publication type |
| 70 | Medina-lezama-2016 | Central arterial hemodynamics of hypertension at high altitude: the intervencion trial | Wrong publication type |
| 71 | Berthelsen-2020 | Highs and lows of sympathetic neurocardiovascular transduction: influence of altitude acclimatization and adaptation. | Wrong study design |
| 72 | Novoa-montero-2002 | Rural endemic chronic cardiopathy or common cardiopathy among chagasic and non chagasic people? | Wrong study design |
| 73 | Narvaez-guerra-2017 | Re:"increased cardiometabolic risk and worsening hypoxemia at high altitude" by miele et al. | Wrong study design |
| 74 | Miranda-2012 | Addressing geographical variation in the progression of non-communicable diseases in peru: the cronicas cohort study protocol | Wrong study design |
| 75 | Vega-2018 | New blood pressure levels in peruvian high altitude populations and the new north american high blood pressure guidelines. | Wrong study design |
| 76 | Moore-2000 | Comparative aspects of high-altitude adaptation in human populations | Wrong study design |
| 77 | Miranda-2009 | The effect on cardiovascular risk factors of migration from rural to urban areas in peru: peru migrant study | Wrong study design |
| 78 | Narvaez-guerra-2017 | Metabolic syndrome and abdominal obesity criteria for andean populations | Wrong study design |
| 79 | Medina-lezama-2016 | A randomized trial of the effect of antihypertensive therapy at low, intermediate, and high altitude: the intervencion study | Wrong study design |
| 80 | Penuela-2015 | Ambulatory blood pressure monitoring profile in population of untreated hypertensive patients of the andes of venezuela | Wrong study design |
| 81 | Luis-2011 | Hypertension, smoking, diabetes and hypercholesterolemia in altitude | Wrong study design |
| 82 | Régulo-2006 | Epidemiología de la hipertensión arterial en el perú | Duplicate population |
| 83 | Rodriguez - 2022 | Clinical characteristics and mortality associated with covid-19 at high altitude: a cohort of 5161 patients in bogotã¡, colombia | Wrong outcome |
| 84 | Brito - 2022 | Metabolic syndrome and risk factors in the adult population | Wrong outcome |
| 85 | Baldeón - 2021 | Prevalence of metabolic syndrome and diabetes mellitus type-2 and their association with intake of dairy and legume in andean communities of ecuador | Duplicate population |
| 86 | Díaz-lazo - 2021 | Clinical and epidemiological characteristics of patients with covid-19 at a high-altitude hospital | Wrong outcome |
| 87 | Moya-salazar - 2021 | Risk factors in rural andean population with covid-19: a retrospective cohort study | Wrong outcome |
| 88 | Vega - 2021 | Systemic blood pressure in peruvian high-altitude populations | Duplicate population |
| 89 | Hernandez-vasquez - 2022 | Association between altitude and the framingham risk score: a cross-sectional study in the peruvian adult population | Wrong outcome |
| 90 | Oliveros - 2021 | Basan index (body mass index, age, sex, arterial hypertension and neck circumference) predicts severe apnoea in adults living at high altitude | Wrong outcome |
| 91 | Simbana-rivera - 2022 | High-altitude is associated with better short-term survival in critically ill covid-19 patients admitted to the icu | Wrong outcome |
| 92 | Bernabe-ortiz - 2021 | Association between body mass index and blood pressure levels across socio-demographic groups and geographical settings: analysis of pooled data in peru | Wrong population |
| 93 | Gudino-gomezjurado - 2021 | Survival analysis of patients with heart failure in the ecuadorian andean population. | Wrong outcome |
| 94 | Calderon-asenjo - 2022 | Association between emotional eating, sociodemographic characteristics, physical activity, sleep duration, and mental and physical health in young adults. | Wrong outcome |
| 95 | García - 2022 | Lifestyle school-based intervention to increase the proportion of adolescents free of components of the metabolic syndrome in an andean region of peru | Wrong outcome |
| 96 | Mejia - 2021 | Cognitive impairment in urban and rural populations of the middle altitude in bolivia: prevalence and associated factors | Wrong outcome |
| 97 | Marmanillo-mendoza - 2021 | Sato2/fio2 rate versus pao2/fio2 rate for predicting mortality in patients with covid-19 in a high-altitude hospital | Wrong outcome |
| 98 | Jibaja - 2022 | Effect of high altitude on the survival of covid-19 patients in intensive care unit: a cohort study. | Wrong outcome |
| 99 | Santos-martínez - 2021 | [deterioration of gas exchange in subjects with an increase in body mass index at an altitude of 2,240 meters above sea level]. | Wrong outcome |
| 100 | Bravo-jaimes - 2021 | Effect of altitude on mortality of end-stage renal disease patients on hemodialysis in peru | Wrong outcome |
| 101 | Galdeano - 2021 | Evaluation of cardiovascular variables in a calchaquã­ population in the middle and high mountains of tucumã¡n | Wrong outcome |
