# Supplementary material for: Prevalence of hypertension in adults living at altitude in Latin America and the Caribbean: A systematic review and meta-analysis
Source: PLoS One. 2023 Oct 12;18(10):e0292111. doi: 10.1371/journal.pone.0292111 (PMC10569637; doi:10.1371/journal.pone.0292111)
Supplement: S4 Table — (DOCX) [file pone.0292111.s005.docx]

| **Supplementary material 5**. Risk of bias of included studies using the Joanna Briggs Institute Critical Appraisal Tool for prevalence studies. | | | | | | | | | |
| --- | --- | --- | --- | --- | --- | --- | --- | --- | --- |
| Study id | Was the sample frame appropriate to address the target population? | Were study participants sampled in an appropriate way? | Was the sample size adequate? | Were the study subjects and the setting described in detail? | Was the data analysis conducted with sufficient coverage of the identified sample? | Were valid methods used for the identification of the condition? | Was the condition measured in a standard, reliable way for all participants? | Was there appropriate statistical analysis? | Was the response rate adequate, and if not, was the low response rate managed appropriately? |
| Bernabe-Ortiz - 2022 | Yes | Yes | Yes | Yes | Yes | No | Yes | No | Yes |
| Segura Vega - 2021 | Yes | Unclear | Yes | Yes | Yes | No | Yes | No | Yes |
| Muñoz - 2021 | No | No | No | Yes | No | No | Yes | Yes | Yes |
| Chambergo-Michilot - 2021 | Yes | Yes | Yes | No | Yes | No | Yes | No | Yes |
| Galdeano - 2021 | Yes | No | Yes | Yes | Yes | No | Yes | Yes | Unclear |
| Diaz-Lazo - 2021 | No | Yes | Yes | Yes | No | Yes | Unclear | Yes | Unclear |
| Pérez-Galarza - 2021 | Yes | Yes | Yes | Unclear | No | No | Unclear | Yes | Yes |
| Menecier - 2021 | Yes | No | No | Yes | No | No | Yes | No | Yes |
| Seclén - 2020 | Yes | Yes | Yes | Unclear | Unclear | No | Yes | No | Yes |
| Bilo – 2020 | Yes | No | No | Yes | Yes | No | Yes | No | Yes |
| Felix - 2020 | Yes | Yes | Yes | Unclear | No | Yes | Yes | Yes | Yes |
| Sosa – 2020 | No | No | No | Yes | Yes | No | Unclear | No | No |
| Mamani - 2019 | No | Yes | Yes | Yes | Yes | Yes | Yes | Yes | Yes |
| Nieto - 2018 | Yes | Yes | Yes | Yes | No | Yes | Yes | Yes | No |
| Bernabé-Ortiz - 2017 | Yes | Yes | Yes | Yes | Yes | No | Yes | Yes | Yes |
| Hernández - 2017 | Yes | Yes | No | Yes | No | Yes | Unclear | No | Yes |
| Ninatanta - 2016 | No | Yes | Yes | Yes | Yes | No | No | Yes | Yes |
| Burroughs - 2015 | Yes | Yes | Yes | Yes | Unclear | No | Yes | No | Unclear |
| Ojeda - 2014 | No | No | Yes | Yes | No | Yes | Yes | Yes | Yes |
| Medina - 2007 | Yes | Yes | Yes | Yes | Yes | No | Yes | Yes | Yes |
| López - 2007 | Yes | Yes | No | Yes | No | No | Yes | No | Unclear |
| Baracco - 2007 | Yes | Yes | No | Unclear | No | Yes | Unclear | Yes | Yes |
| Bernabé-Ortiz - 2017 | Yes | Yes | No | Yes | No | Yes | Yes | No | Yes |
| Sempen - 2010 | Yes | Yes | No | Unclear | No | No | Unclear | No | Yes |
| Pajuelo - 2012 | Yes | Yes | Yes | Unclear | No | No | Unclear | No | Yes |
| Camacho - 2016 | Yes | Yes | Yes | Yes | No | No | Yes | Yes | No |
| Armaza - 2016 | No | Yes | No | Unclear | No | No | Unclear | No | Yes |
| Hernández - 2019 | Yes | Yes | Yes | Unclear | Yes | No | Yes | Yes | Yes |
| Díaz – 2006 | No | No | Yes | Yes | No | Yes | Yes | No | Yes |
| Santos – 2001 | No | No | No | Yes | Yes | Yes | Yes | Yes | Yes |
